# Integrative Transcriptomic and Metabolomic Insights Into Saline-Alkali Stress Tolerance in Foxtail Millet

**DOI:** 10.3390/plants14111602

**Published:** 2025-05-24

**Authors:** Mengxia Han, Qing Tan, Yulu Yang, Hui Zhang, Xingchun Wang, Xukai Li

**Affiliations:** Hou-Ji Laboratory in Shanxi Province, College of Life Sciences, Shanxi Agricultural University, Taigu 030801, China; mmeng1117@163.com (M.H.); tanqing0222@163.com (Q.T.); yulu97_y@163.com (Y.Y.); huizhang@sxau.edu.cn (H.Z.); wxingchun@sxau.edu.cn (X.W.)

**Keywords:** foxtail millet, metabolomics, saline-alkali stress, transcriptomics

## Abstract

Foxtail millet (*Setaria italica*), a cereal crop in China, is renowned for its resilience to abiotic stresses, including saline-alkali conditions. This study examined the transcriptomic and metabolomic responses of two contrasting foxtail millet varieties, B103 (tolerant) and B323 (sensitive), under saline-alkali stress. Physiological analysis showed that B103 exhibited higher growth parameters and chlorophyll content than B323, highlighting its enhanced tolerance. Transcriptomic analysis identified differentially expressed genes (DEGs) enriched in stress-response pathways such as phenylpropanoid biosynthesis, flavonoid metabolism and calcium signaling. Metabolomic profiling revealed differentially accumulated metabolites (DMs) involved in energy and secondary metabolism, including citrate, fumarate and flavonoids. Integration of DEGs and DMs revealed key gene-metabolite interactions, particularly those involving the nicotinamide compound and three candidate genes *Si9g20070*, *Si7g22360* and *Si5g39810*, for future functional validation, which may contribute to stress adaptation. Dynamic clustering of gene expression trends highlighted the importance of rapid stress responses. These findings establish a molecular framework for understanding saline-alkali stress tolerance and provide genetic resources for developing stress-resilient foxtail millet varieties.

## 1. Introduction

Foxtail millet (*Setaria italica*), one of the earliest domesticated crops, holds a significant position as a staple food in China, ranking first among the “five cereals”. It possesses a small diploid genome, rapid growth and diverse germplasm resources, making it an ideal C_4_ model crop for studying stress tolerance [1,2]. *Xiaomi* is an ultra-early-maturing mutant derived from Jingu21, a backbone cultivar in foxtail millet breeding. Beyond its robust genetic foundation, *xiaomi* serves as a C_4_ model plant analogous to Arabidopsis in C_3_ systems, offering unique advantages for studying photosynthesis and stress responses. Saline-alkali stress is a major abiotic factor that significantly reduces global crop yields and impairs the growth and metabolism of many crops [3]. In China, saline soils are widely distributed across the northwestern, northern, northeastern and coastal regions. These soils are rich in soluble salt ions, which accumulate and form a white crust on the surface. Saline soils often clump together, exhibit poor permeability and have low water-holding capacity, severely hindering crop growth [4]. As a major agricultural country, China relies on the stable crop growth to meet its food demands, making the development of saline-alkali-tolerant crops crucial. Foxtail millet exhibits high stress resistance and can thrive under drought and mild saline-alkali conditions. Investigating the molecular mechanisms and metabolic pathways of saline-alkali tolerance in foxtail millet, along with breeding new salt-tolerant varieties, offers a promising strategy for cultivating foxtail millet on saline-alkali soils.

The physiological mechanism by which plants respond to saline-alkali stress is a complex and delicate process, mainly consisting of four aspects, namely the osmotic regulation system, the reactive oxygen species (ROS) scavenging system, the phytohormone regulation system and the ion homeostasis system.

Plants mainly participate in osmotic regulation under saline-alkali stress through inorganic ions and organic osmolytes (proline, betaine). The miR160a-GmARF16-GmMYC2 module in soybean regulates proline biosynthesis. This process adjusts the cellular osmotic pressure, effectively preventing excessive water loss, thereby improving salt resistance [5].

Under saline-alkali stress conditions, the content of ROS in plants will significantly increase. However, the scavenging system in plants can enable the production and scavenging of ROS to reach a dynamic balance, so as to enhance the plant’s adaptation to adverse conditions. After exogenous IAA was applied to rice under alkali stress, the accumulation of H_2_O_2_ and the content of MDA were significantly decreased, and the activities of CAT, POD and SOD in rice plants were significantly increased [6], indicating that exogenous application of IAA could mitigate the alkali-stress-induced inhibition of plant growth by regulating the ROS scavenging system.

Under saline-alkali stress, plants activate a complex hormone regulation network, including abscisic acid (ABA), jasmonic acid (JA), salicylic acid (SA) and auxin (IAA). ABA is a core signaling molecule for plants to respond to saline-alkali stress, and it coordinates various physiological responses through a complex signal transduction network. Several genes were downregulated in *MtHHO3*-overexpressing Arabidopsis, including the ABA biosynthesis gene *NCED3*, the pathogenesis-related genes *PR1* and *PR2* and the ABA/stress-responsive gene *RD29B*. These results suggest that MtHHO3 participates in plant response to salt stress and the ABA-mediated signaling pathway [7].

Under saline-alkali stress, plants need to maintain the ion balance within cells, especially the balance of Na^+^ and K^+^. The high-affinity K^+^ transporter (HKT) family proteins in rice are divided into two classes according to ion selectivity. OsHKT1;1 (Class I HKT) transports Na^+^ alone, whereas OsHKT2;1 (Class II HKT) exhibits both Na^+^ and K^+^ transport activities, both playing essential roles in maintaining Na^+^/K^+^ homeostasis [8].

In conclusion, plants develop a sophisticated physiological defense system through the coordinated regulation of multiple pathways, enabling them to survive and grow under saline-alkali stress conditions.

Integrated transcriptomic and metabolomic analyses have emerged as critical approaches for deciphering molecular responses to saline-alkali stress. In this study, we performed comparative multi-omics profiling of two foxtail millet varieties with contrasting stress tolerance: the saline-alkali tolerant variety “B103” and the saline-alkali sensitive variety “B323” genotypes. This study aimed to pinpoint the DEGs and DMs associated with saline-alkali tolerance in foxtail millet. The aim of this study was to clarify the underlying molecular mechanisms and metabolic pathways involved in foxtail millet’s saline-alkali tolerance. This study will enhance our understanding of the molecular regulatory networks in foxtail millet under saline-alkali stress, offering a theoretical foundation and genetic resources for breeding new saline-alkali tolerant varieties.

## 2. Materials and Methods

### 2.1. Plant Materials

In this experiment, three foxtail millet materials were used: the saline-alkali tolerant variety B103 (Yazizui), the saline-alkali sensitive variety B323 (Lugu10) and mutant *xiaomi*, all provided by Shanxi Agricultural University. The plants were grown hydroponically for 12 days in an artificial climate chamber with 50,000 lux light, a 16 h light/8 h dark cycle, 23–28 °C temperature and 45–60% relative humidity.

The Hoagland nutrient solution was used as the basic solution for plant cultivation. The saline-alkali treatment solution was prepared by additionally adding a 10 mmol/L saline-alkali mixture (NaHCO_3_:Na_2_CO_3_:Na_2_SO_4_:NaCl = 4:1:4:1) to the Hoagland nutrient solution.

### 2.2. Experimental Design

Selected mature, plump and uniformly sized seeds were immersed in a 2% sodium hypochlorite solution for 15 min for surface disinfection, followed by 4 to 5 rinses with distilled water. Thirty disinfected seeds were evenly placed on a glass Petri dish lined with filter paper and allowed to germinate for 5 days. Three replicates were performed for each control and treatment group during the germination period. The control group received Hoagland’s nutrient solution (pH 5.8–6.0), while the treatment group was treated with the same solution, supplemented with 10 mmol/L saline-alkali mixture (pH 8.2–8.4). Uniformly growing seedlings were selected and transferred to a 96-well black hydroponic box for further cultivation for 7 days. The nutrient solution was replaced every three days to ensure proper nourishment of the plants. After a total of 12 days of cultivation, physiological parameters were measured for both foxtail millet varieties. During this period, samples from the bud stage and seedling (two-leaf-one-heart) stage were collected, snap-frozen in liquid nitrogen and stored at −80 °C for RNA extraction, as well as transcriptomic and metabolomic analyses. We conducted saline-alkali stress treatment on *xiaomi* using a 10 mmol/L saline-alkali solution. The leaves were sampled at 10 min, 30 min, 2 h, 24 h, 48 h and 72 h after the stress treatment and then sent to Novogene Co., Ltd. (Beijing, China) for sequencing.

### 2.3. Measurements of Physio-Biochemical Characteristics of Foxtail Millet Response to Saline-Alkali Stress

Ten seedlings were randomly selected from each hydroponic box, totaling 30 plants, to measure root length, plant height, shoot and root fresh weights. The survival rate per box was recorded, and chlorophyll content was measured with three biological replicates. Approximately 0.1 g of leaf tissue was weighed, minced and placed in a centrifuge tube. Chlorophyll extraction was performed using 95% ethanol at room temperature, with samples incubated in the dark for 24 h to ensure complete dissolution of the pigments. After decolorization, absorbance values at 663 nm and 645 nm were measured using a full-wave microplate reader (A51119700DPC, Thermo Fisher Scientific, Waltham, MO, USA), and chlorophyll content was calculated according to the Arnon formulas [9]:

Chlorophyll a (FW mg/g) = (12.7A663 − 2.69A645) × V/(1000 × W)

Chlorophyll b (FW mg/g) = (22.9A645 − 4.68A663) × V/(1000 × W)

Total chlorophyll (FW mg/g) = (20.2A645 + 8.02A663) × V/(1000 × W)

In the above equations, V denotes the final volume of extracted chlorophyll, and W denotes the fresh weight of the leaf used.

### 2.4. Transcriptomic and Metabolomic Analysis of Foxtail Millet Response to Saline-Alkali Stress

#### 2.4.1. Total RNA Quality Control of Sequencing Samples

The foxtail millet leaf samples at the bud stage and the seedling stage were sent to Novogene Co., Ltd. The company carried out RNA extraction, library construction, quality control and sequence alignment of the transcriptome sequencing data. Raw sequence data were filtered using fastp [10] for quality control, and clean reads were obtained by removing those with more than 10% unrecognizable bases and low-quality reads. Clean reads were aligned to the reference *xiaomi* genome using HISAT2 (2.1.0) [11] to obtain positional and sequence information for the samples. The counts of the genes were obtained through the featureCounts program [12], and the transcripts per kilobase of exon model per million mapped reads (TPM) was calculated using the R language.

#### 2.4.2. Sample Preparation and Metabolic Analysis

Samples were freeze-dried using a freeze-drying centrifuge (FreeZone), then ground into powder with a ball mill (GT300) at 30 Hz for 30 s and sieved through a 100-mesh sieve. An amount of 0.1 g of powder was weighed (three replicates) into a 2 mL centrifuge tube and stored at −80 °C. A total of 1.0 mL of 70% methanol extraction solution with 0.1 mg/L lidocaine (internal standard) was added to the sample tube. The samples were vortexed and immediately stored at 4 °C. After 10 min, the sample was vortexed three times and then left at 4 °C overnight to ensure full reaction. After the overnight reaction, the mixture was centrifuged at 14,000 rpm for 5 min at 4 °C. An amount of 600 μL of the supernatant was transferred to a clean 2 mL tube and centrifuged again at 14,000 rpm for 5 min at 4 °C. The supernatant was transferred through a filter into a new centrifuge tube. An amount of 150 μL of the filtered extract was transferred into a sample vial with an insert for subsequent analysis.

Untargeted metabolite detection was conducted by Huazhong Agricultural University using an ultra-performance liquid chromatography coupled with an electrospray tandem mass spectrometry (UPLC-ESI-MS/MS) system. A quality control sample, consisting of a mix of extracts from several foxtail millet varieties, was inserted between every 10 samples that were analyzed.

#### 2.4.3. Screening and Analysis of Differentially Expressed Genes and Metabolites

The Deseq2 [13] package was used to screen for DEGs in TPM data, with *p* < 0.05 and |log2FoldChange| ≥ 1 set as the thresholds for significant differences in gene expression. DEGs between samples were shown using Venn diagrams. Principal component analysis (PCA) and orthogonal partial least squares discriminant analysis (OPLS-DA) were conducted on metabolome relative content data using R. DMs were identified based on OPLS-DA results. Metabolites with variable importance in projection (VIP) > 1, *p* < 0.05 and |log2FoldChange| ≥ 1 were considered to be differentially accumulated. The DMs between samples were presented using Venn diagrams.

#### 2.4.4. GO and KEGG Enrichment Analysis

Functional annotation and pathway enrichment analysis of the DEGs were performed using the Gene Ontology (GO) database [14,15] (https://geneontology.org/) and KEGG database [16] (https://www.genome.jp/kegg/, accessed on 26 November 2024), aiding in the identification of target genes and key pathways. GO enrichment analysis of the DEGs was conducted using the org.Si.eg.db (v3.0) (https://github.com/guokai8/org.Si.eg.db, accessed on 26 November 2024) package in R, with *p* < 0.05 indicating statistical significance. KEGG pathway enrichment analysis was conducted on the DEGs and DMs, with bubble plots used to display the results.

#### 2.4.5. Profiling the Gene Expression Patterns of Xiaomi Under Saline-Alkali Stress

Mfuzz [17,18], a fuzzy c-means clustering algorithm, effectively identifies dynamic patterns and trends in time series data. To investigate the dynamic changes in gene expression levels in *xiaomi* after saline-alkali stress, the Mfuzz package in R was used for hierarchical clustering of the time-series differentially expressed genes in *xiaomi*. We carried out exploratory analyses by testing different values of the clustering parameter “c”. When c = 16, there was good separation and cohesion within each cluster.

#### 2.4.6. Correlation Network Diagram

A correlation analysis was performed on all DEGs and DMs. DEGs and DMs with Pearson correlation coefficients greater than 0.7 were statistically analyzed, and their correlations were represented by network diagrams.

#### 2.4.7. Metabolic Pathway Analysis

Metabolic pathways of differentially accumulated metabolites in foxtail millet were constructed using the KEGG database. The differential metabolite contents were visualized as heatmaps in R.

## 3. Results

### 3.1. Phenotypic and Physiological Analysis

**Figure 1:**
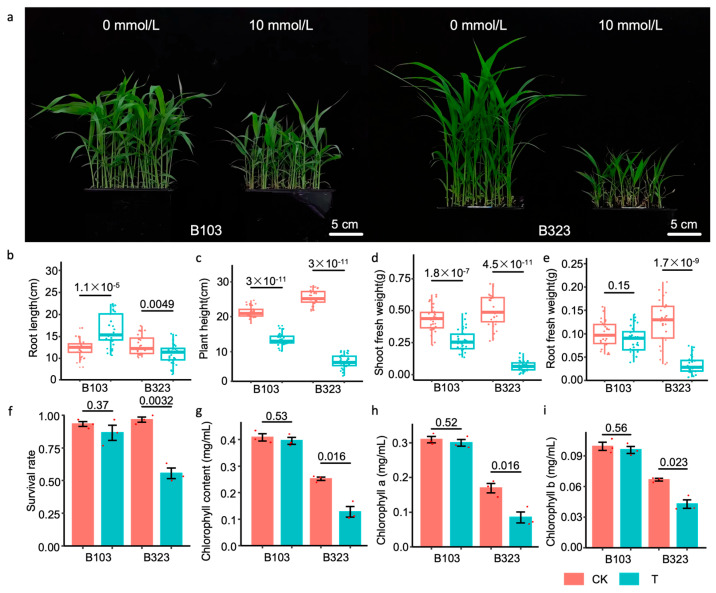
Growth phenotypes and physiological phenotypes of two foxtail millet varieties under saline-alkali stress. (**a**) Growth phenotypes of two foxtail millet varieties under saline-alkali stress. (**b**) Root length. (**c**) Plant height. (**d**) Shoot fresh weight. (**e**) Root fresh weight. (**f**) Survival rate. (**g**) Chlorophyll content. (**h**) Chlorophyll a. (**i**) Chlorophyll b. CK: the control group, 0 mmol/L, Hoagland nutrient solution. T: the treatment group, 10 mmol/L, Hoagland nutrient solution + 10 mmol/L saline-alkali solution. Three replicates were performed for each control and treatment group during the seed germination period. Specific *p*-values for key comparisons are shown above the bars: *p* < 0.05 means the significance level, and *p* < 0.01 means the extreme significance level.

After exposing the salt-tolerant variety B103 and the salt-sensitive variety B323 to saline-alkali stress, we observed growth inhibition in both varieties. However, B103 exhibited significantly greater tolerance to saline-alkali stress compared to B323 (Figure 1a). Specifically, the plant height, shoot fresh weight and root fresh weight of B323 were markedly reduced, with the changes being more pronounced in B323 than in B103. Additionally, while the root length of B323 was significantly decreased, B103 showed an increase in root length under saline-alkali stress (Figure 1b–e).

The statistical analysis of the survival rates revealed no significant difference in the survival rate of B103 between normal and saline-alkali stress conditions. In contrast, the survival rate of B323 under saline-alkali stress was only 55.55% (Figure 1f). Chlorophyll content analysis further supported these findings, showing no significant change in the chlorophyll content of B103 after saline-alkali treatment. However, B323 exhibited a significant reduction in chlorophyll content, suggesting that saline-alkali stress impacts the chlorophyll content of foxtail millet, with a more pronounced effect on the salt-sensitive variety (Figure 1g–i).

### 3.2. Transcriptomic and Metabolomic Analysis

#### 3.2.1. Screening and Analysis of Differentially Expressed Genes and Metabolites

To identify genes involved in the response to saline-alkali stress at the bud and seedling (two-leaf-one-heart) stages in foxtail millet, we constructed four comparison groups between control and saline-alkali treatment conditions: (1) bud stage: B103-B-CK vs. B103-B-T and B323-B-CK vs. B323-B-T, and (2) seedling stage: B103-S-CK vs. B103-S-T and B323-S-CK vs. B323-S-T. Genes with *p* < 0.05 and |log2FoldChange| ≥ 1 were defined as differentially expressed genes.

**Figure 2:**
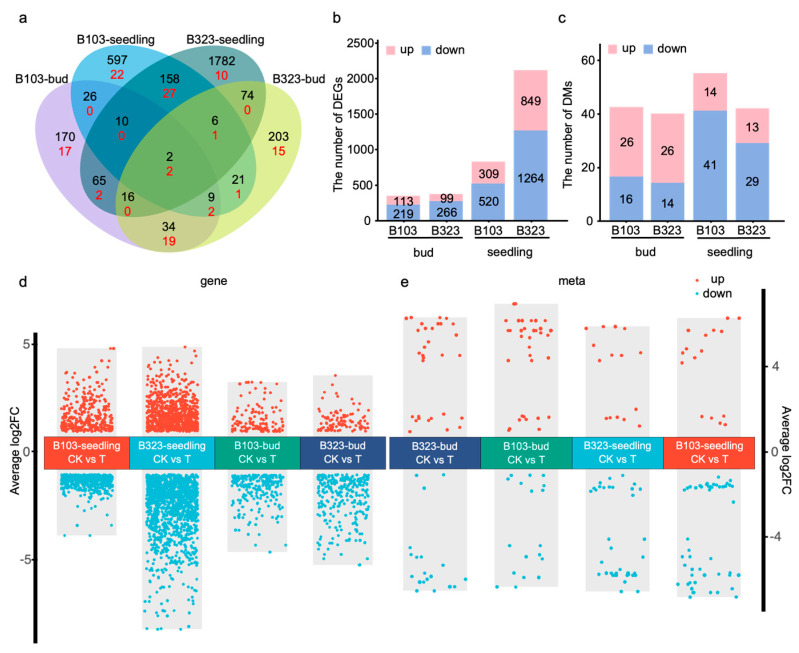
Venn and bar diagram of different expressed genes and metabolites. (**a**) Venn diagram showing the overlap of DEGs and DMs. The number of DEGs is labeled in black. The number of DMs is labeled in red. (**b**) Bar chart of DEGs. (**c**) Bar chart of DMs. (**d**) Comparison of DEGs between different groups. (**e**) Comparison of DMs between different groups.

A Venn diagram was created to show the overlap of DEGs across different stages. At the bud stage, 61 DEGs were shared between the two comparison groups, and 176 DEGs were common between the groups at the seedling stage (Figure 2a). At the bud stage, 332 DEGs were identified in B103-CK/B103-T, with 113 upregulated and 219 downregulated DEGs, while 365 DEGs were identified in B323-CK/B323-T, with 99 upregulated and 266 downregulated DEGs. At the seedling stage, 829 DEGs were identified in B103-CK/B103-T, with 309 upregulated and 520 downregulated DEGs, and 2113 DEGs were identified in B323-CK/B323-T, with 849 upregulated and 1264 downregulated DEGs (Figure 2b). These results suggest that a substantial number of genes are involved in the response to saline-alkali stress in both saline-alkali tolerant (B103) and sensitive (B323) varieties. Notably, the number of DEGs was greater at the seedling stage than at the bud stage, and more DEGs were found in the saline-alkali sensitive variety (B323) than in the tolerant variety (B103), indicating that the response of sensitive varieties to saline-alkali stress is more pronounced (Figure 2d).

OPLS-DA was performed to analyze DMs at both the bud and seedling stages. In the OPLS-DA models, R2X and R2Y values represent the explanatory power of the model for the X and Y matrices, respectively, and Q2 reflects the model’s predictive ability. All comparison groups had a Q2 value greater than 0.5, indicating that the constructed model was appropriate. The R2X and R2Y values at the bud stage were 0.348 and 0.996, respectively, and at the seedling stage, they were 0.329 and 0.997 (Figure S1a,d). A permutation test was performed to check for overfitting, and the results indicated no overfitting, as the R2Y and Q2Y values (represented by scatter points) were lower than the true values (horizontal line) (Figure S1b,e). Principal component analysis (PCA) of DMs revealed that the first and second principal components (PC1 and PC2) contributed 13.59% and 9.49% of the variance, respectively. The control and treatment groups were clearly separated along PC1 in both stages (Figure S1c,f), which aligns with the observed phenotypic differences. This suggests that the DMs between B103 and B323 changed markedly after saline-alkali stress, in line with the observed stress response.

Based on the OPLS-DA results, fold change and VIP values were used to identify DMs. Metabolites with |log2FoldChange| ≥ 1 and VIP > 1 were considered differentially accumulated. At the bud stage, 42 DMs were identified in B103-CK/B103-T, with 26 upregulated and 16 downregulated DMs, and 40 DMs were identified in B323-CK/B323-T, with 26 upregulated and 14 downregulated DMs. At the seedling stage, 55 DMs were identified in B103-CK/B103-T, with 14 upregulated and 41 downregulated DMs, and 42 DMs were identified in B323-CK/B323-T, with 13 upregulated and 29 downregulated DMs (Figure 2c). According to the magnitude of the fold difference, Figure 2e shows the up- and downregulation of DMs across different groups.

#### 3.2.2. Transcription Factor Analysis Under Saline-Alkali Stress

Transcription factors (TFs) are crucial regulatory elements that control gene expression and play a vital role in plant growth, development and stress responses [19]. To investigate the transcriptional regulation in foxtail millet under saline-alkali stress, DEGs identified at the bud and seedling stages were subjected to transcription factor prediction (Table S1). At the bud stage, 48 DEGs were annotated to 16 TF families, including WRKY, MYB, bZIP, bHLH, NAC, B3 and GRAS. At the seedling stage, 91 DEGs were assigned to 32 TF families, including ERF (ethylene-responsive factor), WRKY, MYB, bHLH, AP2 (APETALA2), ARF (auxin response factor) and bZIP. Notably, ERF, AP2 and RAV (related to ABI3/VP) are subfamilies within the AP2/ERF transcription factor family, which primarily regulate gene expression through the cis-acting dehydration-responsive element/C-repeat (DRE/CRT) element [20]. These TFs play significant roles in regulating plant responses to abiotic stresses. In addition, WRKY [21], MYB [22], bZIP [23] and NAC [24] transcription factors are also involved in various abiotic stress response pathways. The analysis highlights differences in the TFs activated in response to saline-alkali stress at different growth stages, indicating stage-specific regulatory mechanisms.

#### 3.2.3. GO and KEGG Enrichment Analysis of Differentially Expressed Genes and Differential Metabolites

For functional analysis, DEGs were subjected to GO annotation at the bud and seedling stages, followed by an analysis of the top 50 annotated pathways. The results indicated that all 50 significantly enriched GO terms were associated with biological processes (BP). The functional enrichment of DEGs under saline-alkali stress varied between the different foxtail millet varieties. At the bud stage, the top four enriched GO terms, based on the number of genes, were as follows: response to stimulus (GO:0050896), response to stress (GO:0006950), response to chemicals (GO:0042221) and defense response (GO:0006952). Notably, the GO terms “response to stress” (GO:0006950), “response to stimulus” (GO:0050896) and “defense response” (GO:0006952) were significantly enriched, indicating that foxtail millet initiates specific responses to saline-alkali stress at the bud stage (Figure S2a). At the seedling stage, the top four enriched GO terms were as follows: response to stimulus (GO:0050896), carbohydrate metabolic process (GO:0005975), lipid metabolic process (GO:0006629) and polysaccharide metabolic process (GO:0005976). Among these, the “carbohydrate metabolic process” (GO:0005975) was the most significantly enriched term. Other enriched GO terms included “secondary metabolite biosynthetic process” (GO:0044550) and “carbohydrate metabolic process” (GO:0044262) (Figure S2b).

**Figure 3:**
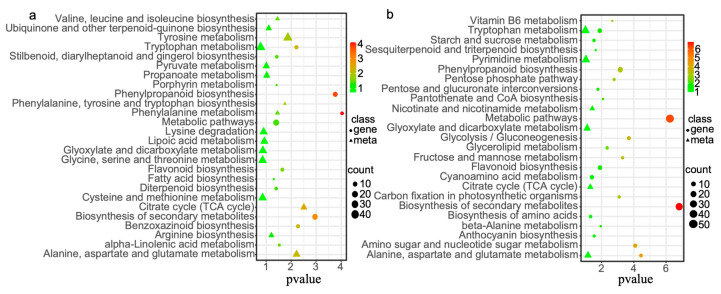
KEGG pathway of differential genes and metabolites. (**a**) KEGG pathway of DEGs and DMs at the bud stage. (**b**) KEGG pathway of DEGs and DMs at the seedling stage. Circles represent DEGs and triangles represent DMs.

To further analyze the DEGs and DMs associated with the saline-alkali tolerance of foxtail millet, we conducted KEGG pathway analysis on the DEGs and annotated DMs. At the bud stage, only 11 metabolites had corresponding entries in the KEGG database, while at the seedling stage, only seven metabolites were present in the KEGG database (Table S2). We visualized the results using bubble plots to highlight pathways with the smallest significant Q-values. The DEGs at the bud stage were enriched in 12 KEGG pathways. The top three most enriched pathways were metabolic pathways, biosynthesis of secondary metabolites and phenylpropanoid biosynthesis. Enrichment analysis of DMs showed that at the bud stage, DMs were primarily annotated in pathways such as the citrate cycle (TCA cycle), alanine, aspartate and glutamate metabolism, tyrosine metabolism, phenylalanine, tyrosine and tryptophan biosynthesis, valine, leucine and isoleucine biosynthesis and phenylalanine metabolism (Figure 3a). The DEGs at the seedling stage were enriched in 21 KEGG pathways, with 52 genes enriched in metabolic pathways, 38 genes enriched in biosynthesis of secondary metabolites and 17 genes enriched in phenylpropanoid biosynthesis. At the seedling stage, DMs were annotated and enriched in pathways including nicotinate and nicotinamide metabolism, citrate cycle (TCA cycle), alanine, aspartate and glutamate metabolism, glyoxylate and dicarboxylate metabolism, pyrimidine metabolism and tryptophan metabolism (Figure 3b). Some metabolic pathways, such as the citrate cycle (TCA cycle), alanine, aspartate and glutamate metabolism and glyoxylate and dicarboxylate metabolism, overlapped between the bud and seedling stages.

#### 3.2.4. Correlation Network of Differentially Expressed Genes and Differential Metabolites

**Figure 4:**
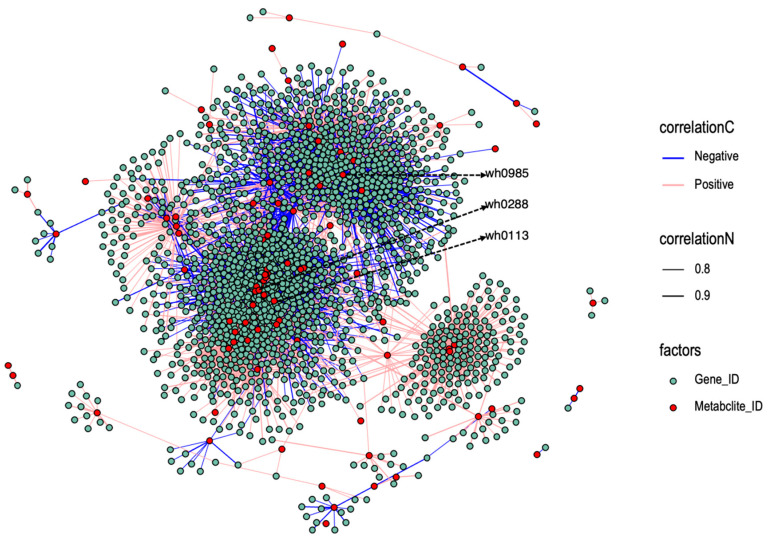
Correlation network diagram of all differential expressed genes and differential accumulated metabolites. The correlation coefficient is greater than 0.7. Pink edges indicate positive correlations; blue edges indicate negative correlations. Gene_IDs are displayed as red circular nodes, with metabolite_IDs depicted as green circular nodes.

A correlation network analysis was performed between all selected DEGs and DMs. A network diagram was constructed for gene-metabolite pairs with a correlation coefficient greater than 0.7 (Figure 4). The results revealed that 3032 gene-metabolite pairs exhibited a positive correlation in their differential expression patterns, while 3264 pairs showed a negative correlation. Notably, the metabolite with the highest number of correlations is 4-[(Butylcarbamoyl)amino]-1-ethyl-1H-pyrazole-5-carboxamide (wh0985), which is correlated with a total of 375 DEGs. In the differentially accumulated metabolites, 1-methyladenine (wh0288) present in the KEGG database is significantly correlated with 224 DEGs, and nicotinamide (wh0113) is significantly correlated with 73 DEGs.

Nicotinamide is an important metabolic substance. We annotated 73 genes related to nicotinamide and found that *Si9g20070* was enriched in nicotinamide adenine dinucleotide phosphate (NADPH) dehydrogenase activity (GO:0003959). NADPH dehydrogenase (NDH) is involved in cyclic electron transport (CET), which plays a significant role in regulating photosynthetic machineries and responding to adverse environmental conditions [25]. *Si7g22360* and *Si5g39810* were annotated as members of the MYB transcription factor family. MYB transcription factors are a typical family of transcription factors closely associated with abiotic stresses. Therefore, these genes are important candidate genes.

#### 3.2.5. The Gene Expression Patterns of Xiaomi

**Figure 5:**
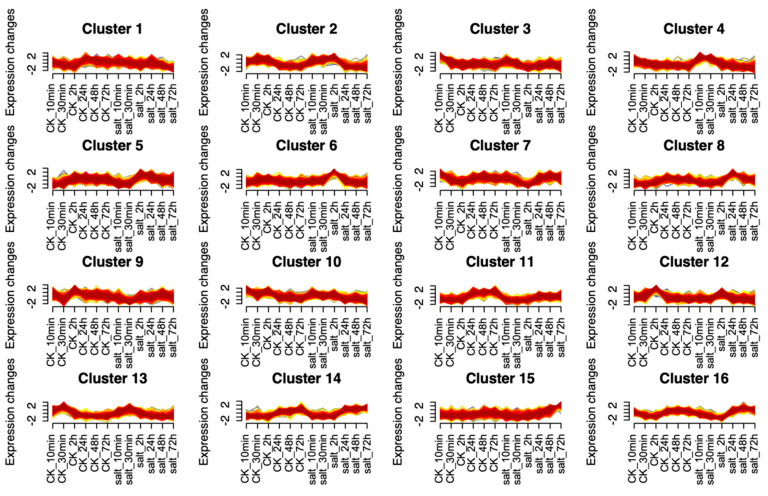
Mfuzz analysis of *xiaomi* at different saline-alkali treatment times. Mfuzz is based on fuzzy c-means clustering (c = 16) of transcriptomic dynamics during stress treatment (0–72 h). CK: the control group, Hoagland nutrient solution. Salt: the treatment group, Hoagland nutrient solution + 10 mmol/L saline-alkali solution.

To investigate the dynamics of gene expression in *xiaomi* under different saline-alkali treatment durations, we performed Mfuzz analysis on transcriptomic data collected at various time points. As shown in Figure 5, the genes were divided into 16 modules. Differentially expressed genes can be detected 10 min after saline-alkali treatment, suggesting that *xiaomi* responded to stress rapidly. Among these, genes in cluster 5 and cluster 6 exhibited an expression pattern characterized by an initial increase followed by a decrease after saline-alkali stress, with the highest expression levels observed at 2 h. GO enrichment analysis of genes from cluster 5 and cluster 6 revealed that a substantial number of genes in both groups were mainly enriched in response to stimulus (GO:0050896) (Figure S3), which further elucidates a rapid response to saline-alkali stress. Among these, cluster 5 shows significant enrichment in zinc ion transmembrane transport, protein-DNA complex organization/assembly and nucleosome organization/assembly. Cluster 6 exhibits significant enrichment in the L-arginine biosynthetic process and glutamine family amino acid biosynthetic process.

#### 3.2.6. Analysis of Significant Biological Pathway Changes

**Figure 6:**
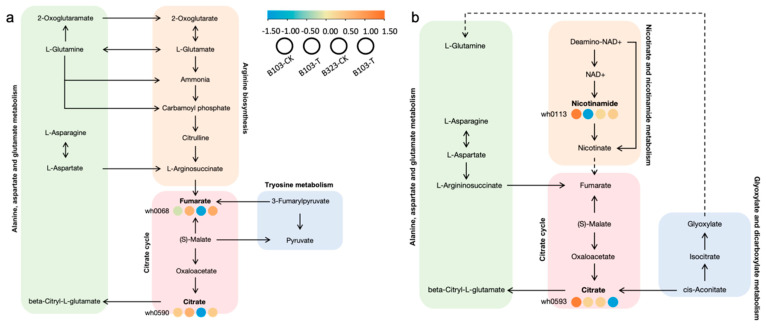
Metabolic pathways of two foxtail millet varieties under saline-alkali stress. (**a**) The important metabolic pathways at the bud stage. (**b**) The important metabolic pathways at the seedling stage.

At the bud stage, the KEGG pathways that were enriched include alanine, aspartate and glutamate metabolism, arginine biosynthesis, the citrate cycle (TCA cycle) and tyrosine metabolism. Among these, the metabolites fumarate (wh0068) and citrate (wh0590), which are part of the citrate cycle, showed elevated levels in both varieties, with a more pronounced increase in B323. This suggests that B323 responds to saline-alkali stress by adjusting its respiration to consume energy (Figure 6a). At the seedling stage, the enriched pathways included alanine, aspartate and glutamate metabolism, nicotinate and nicotinamide metabolism, the citrate cycle (TCA cycle) and glyoxylate and dicarboxylate metabolism. In these pathways, the content of citric acid (wh0593) in the citrate cycle decreased in both varieties. Notably, in the nicotinate and nicotinamide metabolism pathway, the level of nicotinamide (wh0113) was significantly lower in B103 compared to B323 after saline-alkali stress (Figure 6b). This difference may be attributed to saline-alkali stress inducing iron deficiency in B323.

## 4. Discussion

### 4.1. Integrated Multi-Omics Reveals Foxtail Millet’s Saline-Alkali Stress Response

Integrated transcriptomic and metabolomic analyses have become essential tools for studying plant responses to saline-alkali stress in recent years. For instance, a multi-omics approach explored the complex response mechanisms of two alfalfa species under combined cold and saline-alkali stress. This study found that the tricarboxylic acid cycle (TCA) in both varieties responded positively to the stress, with significant differences in gene expression and flavonoid content in the flavonoid biosynthesis pathway [26]. Using integrated transcriptomics and metabolomics analyses, the molecular mechanism of salt tolerance in upland rice landrace 17SM-19 was investigated. Comparative analysis of genes and metabolites before and after salt stress induced growth inhibition revealed that rice salt tolerance involves osmotic regulation, ion homeostasis and ROS scavenging [27].

This study provides a comprehensive multi-omics approach to uncover the molecular and metabolic adaptations of foxtail millet under saline-alkali stress. As depicted in Figure 3, KEGG enrichment analysis revealed that foxtail millet saline-alkali tolerance involved phenylpropanoid biosynthesis, flavonoid metabolism and calcium signaling. Phenylpropanoid biosynthesis is a key metabolic pathway influenced by phytohormones, as well as biotic and abiotic stresses [28]. Lignin and flavonoids represent major branches of this pathway. The transcriptional upregulation of lignin biosynthetic genes promotes lignin deposition and secondary cell wall thickening and enhances tolerance to salt and osmotic stress in apple [29]. Flavonoids act as antioxidants, scavenging ROS and protecting plants from abiotic stress-induced damage [30]. Overexpression of *MsFLS13* in alfalfa enhances flavonol synthesis, thereby improving saline-alkali stress tolerance [31].

Metabolomic profiling revealed significant DMs involved in energy and secondary metabolism. The activation of the TCA cycle generates energy for organic acid production and pH homeostasis [32]. Metabolites such as citrate and fumarate were elevated in both varieties, with higher accumulation in B323, indicating increased respiration to meet energy demands under stress. However, the reduced nicotinamide levels in B103 suggest a more efficient metabolic adjustment.

Integrated transcriptomic and metabolomic data revealed gene-metabolite interactions that underpin saline-alkali tolerance. The identification of stage-specific gene expression trends using Mfuzz revealed rapid and dynamic stress responses in foxtail millet. Additionally, correlation network analysis emphasized the importance of nicotinamide metabolism and phenolic compound synthesis in mitigating stress effects, and a key candidate gene was screened out.

### 4.2. Regulatory Mechanisms of TF Families in Abiotic Stress Responses

TFs are the key regulators of plant responses to stress. Transcription factors such as AP2/ERF [20], WRKY [21], MYB [22], bZIP [23] and NAC [24] are involved in response to most abiotic stresses. The AP2/ERF transcription factor family comprises multiple subfamilies such as ERF, AP2 and RAV. This study found that the AP2/ERF family is the TF family with the largest number of members involved in saline-alkali stress responses during both the bud and seedling stages (Table S1). OsERF52 directly regulates the expression of *C-Repeat Binding Factor* (*CBF*) in rice to respond to low-temperature stress [33]. The WRKY transcription factor WRKY57 in tomato acted as a negative regulator in salt stress response by directly attenuating the transcription of salt-responsive genes (*SlRD29B* and *SlDREB2*) and an ion homeostasis gene (*SlSOS1*) [34]. Overexpression of MYB12 and MYB75 in transgenic plants significantly increased the accumulation of flavonoids with strong antioxidant activity, thus enhancing the tolerance of Arabidopsis to abiotic stresses such as drought and oxidative stresses [35]. In particular, the transcription factor bZIP44 positively regulated the transcriptional activity of *nicotianamine synthase* (*NAS*) genes *NAS2* and *NAS4* through interaction with MYB10 and MYB72, which promoting nicotianamine (NA) synthesis and improving Fe transport [36]. These findings reveal that different TFs form complex regulatory networks by directly regulating the expression of stress-related genes or interacting with other factors, collaboratively mediating plants’ adaptive responses to abiotic stresses such as saline-alkali, low temperature, drought, oxidative stress and iron deficiency. This provides important targets and theoretical foundations for molecular breeding of stress-resistant crops.

### 4.3. Nicotinamide Plays a Crucial Role in Saline-Alkali Stress Responses

Under saline-alkali stress, plants can dynamically regulate cellular ion homeostasis through selective ion uptake, efflux and compartmentalization processes. NAS genes catalyze the synthesis of nicotinamide; nicotinamide not only engages in the transport, distribution and storage of iron ions in plants but also mediates the transport of other metal ions, thereby enhancing the plant’s tolerance to metals [37]. Therefore, this substance may be a potential factor contributing to its stress tolerance. The nicotinamidase gene *AtNIC1* has been confirmed as one of the key enzymes catalyzing the conversion of nicotinamide to nicotinic acid in *Arabidopsis thaliana*. The *nic1-1* mutant plants have lower levels of NAD and NADP and exhibit hypersensitivity to treatments of abscisic acid and NaCl [38]. In this study, nicotinamide (wh0113) belongs to the DMs of nicotinate and nicotinamide metabolism pathways. Due to the extremely low solubility of iron ions in saline-alkali soils, it causes iron deficiency stress in plants [39]. Therefore, nicotinamide (wh0113) serves as a precursor for phytosiderophores, which facilitate iron transport in plants.

Through the annotation of genes associated with nicotinamide, we identified three candidate genes. *Si9g20070* was enriched in nicotinamide adenine dinucleotide phosphate (NADPH) dehydrogenase activity (GO:0003959). NADPH is a universal electron donor that provides the reductant power for generating antioxidant molecules, for example, reduced glutathione (GSH), a main player in regulating the redox level of cells [40]. Additionally, NADPH is the driving power for NADPH oxidases (Nox) to produce ROS. Cytosolic NADP-dehydrogenases regulate ROS homeostasis by controlling NADPH regeneration [41]. NDH-mediated cyclic electron transport can promote ATP synthesis, provide energy for the antioxidant system, reduce ROS accumulation and play a crucial role in plant stress resistance [42]. Therefore, *Si9g20070* may enhance the saline-alkali tolerance of plants by maintaining ROS homeostasis. *Si7g22360* and *Si5g39810* were annotated as members of the MYB transcription factor family. MYB proteins are key factors in regulatory networks controlling development, metabolism and responses to biotic and abiotic stresses. However, the regulatory mechanisms of these genes in saline-alkali stress responses require further in-depth exploration through molecular biology experiments and functional validation.

Saline-alkali environments are often accompanied by changes in many metabolic processes and metabolites. The enriched pathways and key candidates identified present valuable targets for genetic improvement. These findings enhance our understanding of saline-alkali stress tolerance mechanisms and provide a solid foundation for breeding stress-tolerant foxtail millet varieties. Future research should functionally validate these candidate genes and pathways to further enhance stress resilience in crops.

## 5. Conclusions

This study provides detailed insights into the molecular and metabolic mechanisms underlying saline-alkali stress tolerance in foxtail millet (*Setaria italica*). Using integrated transcriptomic and metabolomic analyses of two contrasting varieties, B103 (tolerant) and B323 (sensitive), we identified key pathways, metabolites and candidate genes involved in stress adaptation. Physiological evaluations demonstrated that B103 exhibited superior growth and photosynthetic efficiency under saline-alkali stress, highlighting its potential as a genetic resource for improving stress tolerance. Transcriptomic analysis revealed differentially expressed genes (DEGs) enriched in phenylpropanoid biosynthesis, flavonoid metabolism and calcium signaling pathways, all of which are critical for stress mitigation. Metabolomic profiling emphasized the roles of energy metabolism and secondary metabolites, including flavonoids and nicotinamide in maintaining cellular homeostasis during stress. Integrated multi-omics data uncovered key gene-metabolite interactions and identified an important compound nicotinamide and three candidate genes *Si9g20070*, *Si7g22360* and *Si5g39810*, providing new insights into dynamic stress responses.

Our findings enhance the understanding of saline-alkali stress tolerance mechanisms in foxtail millet and establish a foundation stress-resilient crops through genetic engineering and breeding. Future research should focus on the functional validation of the identified genes and metabolites to further enhance crop stress tolerance, especially in salinity- and alkalinity-affected regions.

## Data Availability

The raw RNA-seq sequencing data from our transcriptome experiment are also available in the BIG Data Center under accession number PRJCA035053.

## Supplementary Materials

The following supporting information can be downloaded at https://www.mdpi.com/article/10.3390/plants14111602/s1. Figure S1: OPLS-DA score plot and permutation test; Figure S2: The significantly enriched GO term of differential genes in the bud stage and the seedling stage; Figure S3: The significantly enriched GO term of differential genes in the *xiaomi*. Table S1: Transcription factors in differentially expressed genes at the bud and seedling stage. Table S2: Differentially accumulated metabolites at the bud and seedling stage.
