# Supplementary material for: Integrative Transcriptomic and Metabolomic Insights Into Saline-Alkali Stress Tolerance in Foxtail Millet
Source: Plants (Basel). 2025 May 24;14(11):1602. doi: 10.3390/plants14111602 (PMC12158200; doi:10.3390/plants14111602)
Supplement: Supplementary file 1 [file plants-14-01602-s001.zip › plants-3571858-supplementary.pdf]

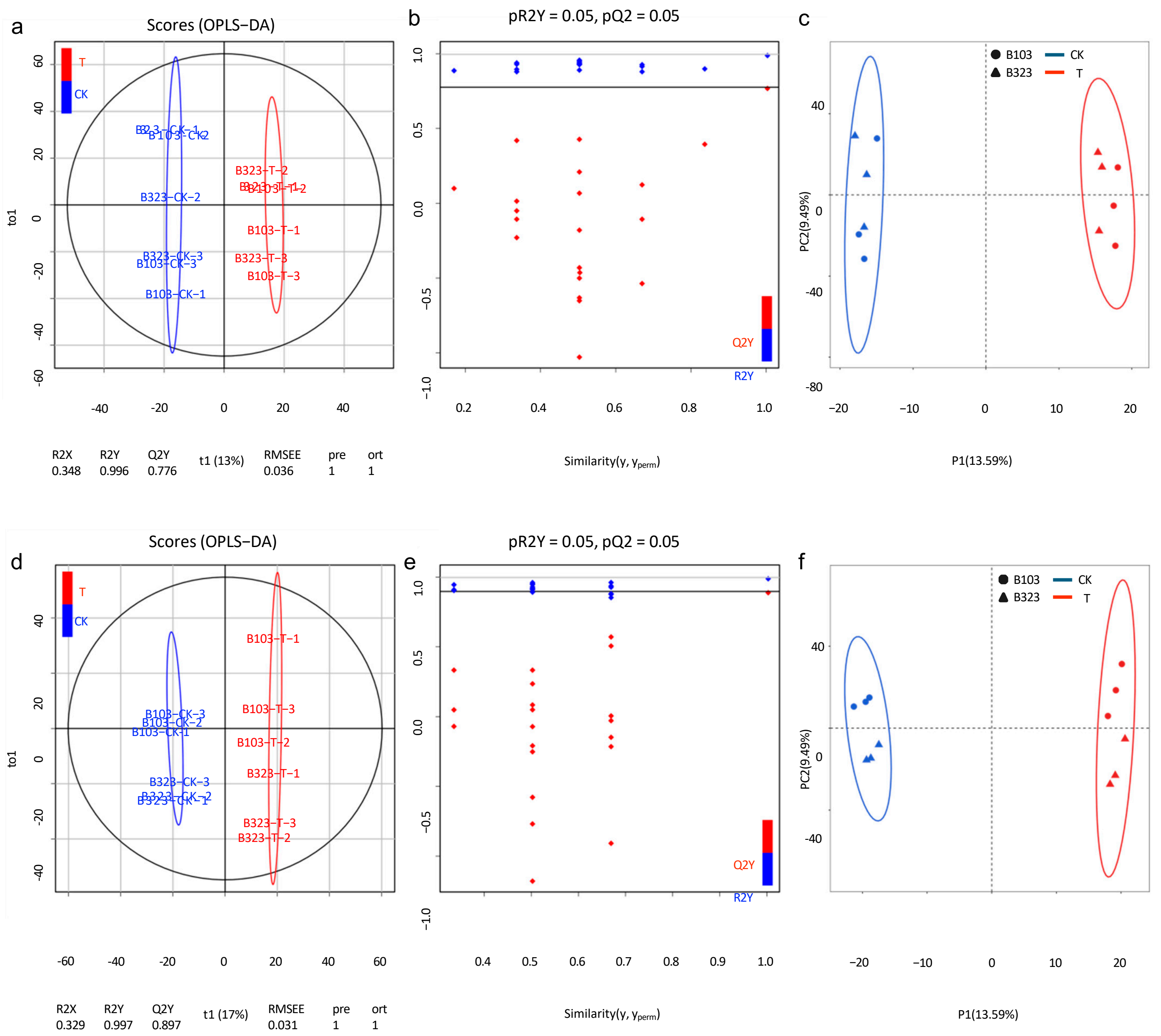

Figure S1 OPLS-DA score plot and permutation test. **a-b** OPLS-DA model at the bud stage. **c** PCA score plots at the bud stage. **d-e** OPLS-DA model at the seedling stage. **f** PCA score plots at the seedling stage.

Table S1 Transcription factors in differentially expressed genes at the bud and seedling stage

	TF	Gene number		TF	Gene number	TF	Gene number
bud stage	ERF	10	seedling stage	bHLH	19	DBB	3
	WRKY	7		ERF	18	GRF	3
	bZIP	6		MYB	18	YABBY	3
	HSF	5		WRKY	17	AP2	2
	MYB_related	4		NAC	15	ARF	2
	bHLH	3		HD-ZIP	13	CPP	2
	NAC	2		TALE	12	GATA	2
	LBD	2		bZIP	10	SBP	2
	B3	1		G2-like	7	TCP	2
	CAMTA	1		HSF	6	BES1	1
	C2H2	1		Dof	5	EIL	1
	C3H	1		C2H2	4	FAR1	1
	GRAS	1		C3H	4	LBD	1
	HD-ZIP	1		CO-like	4	NF-YC	1
	MIKC_MADS	1		MIKC_MADS	4	RAV	1
	MYB	1		MYB_related	4		
	Trihelix	1		Trihelix	4		

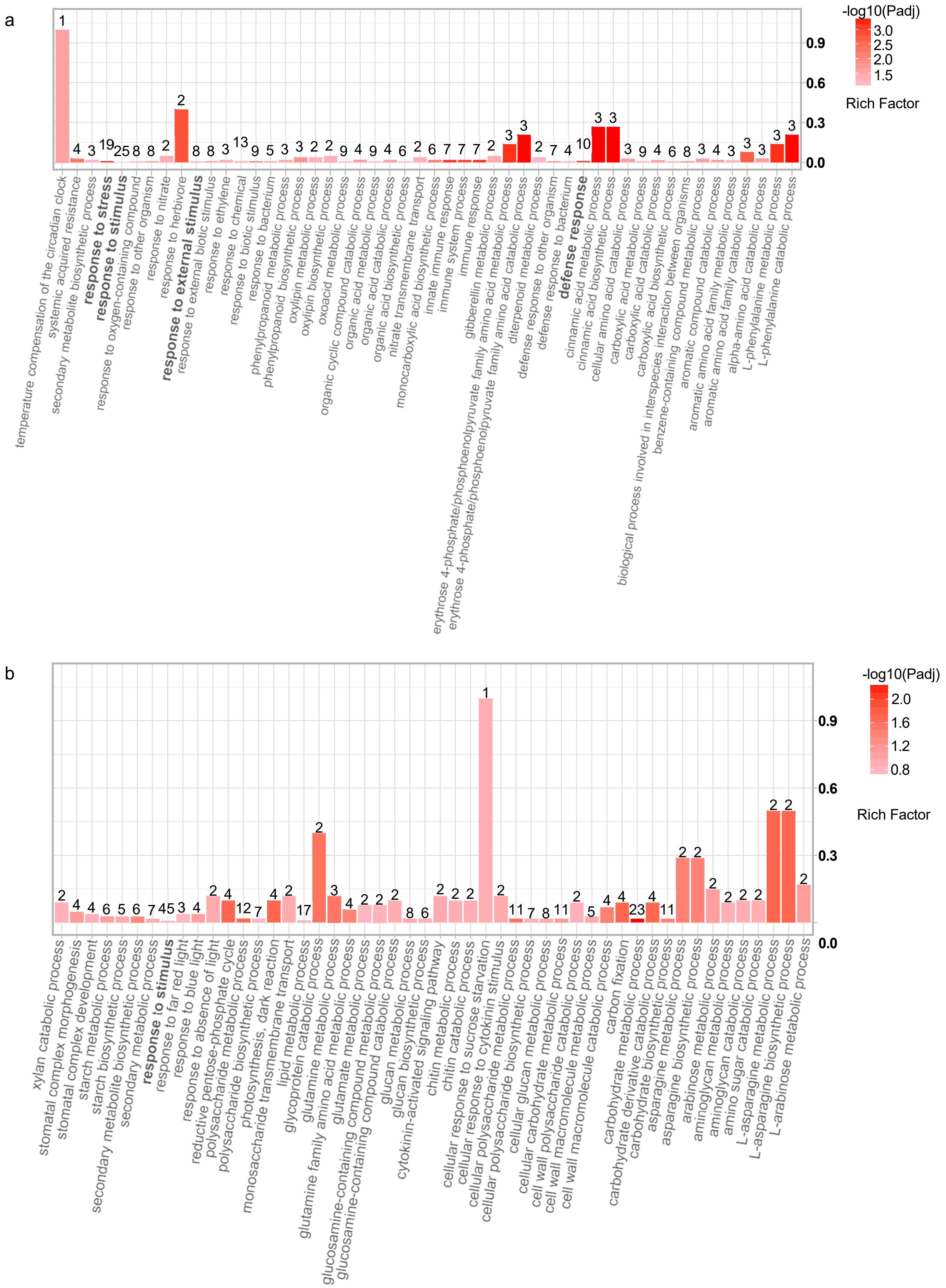

Figure S2 The significantly-enriched GO term of DEGs in the bud stage and the seedling stage. **a** The significantly-enriched GO term of DEGs at the bud stage. **b** The significantly-enriched GO

term of

DEGs at the seedling stage.

Table S2 Differentially accumulated metabolites at the bud and seedling stage

	ID	Name		ID	Name
	wh0010	2-Oxobutyric acid		wh0496	5,7-Dihydroxychromone
	wh0468	7-Methoxycoumarin		wh0126	Thymine
	wh0523	L-Tyrosine		wh0462	Indole-3-acetic acid
	wh0068	Fumaric acid		wh0113	Nicotinamide
	wh0590	Citric acid		wh0593	Citric acid
bud stage	wh1131	L-Saccharopine	seedling stage	wh0288	1-Methyladenine
	wh0244	N-Nitrosoproline		wh0973	Muramic acid
	wh0362	Allicin			
	wh0462	Indole-3-acetic acid			
	wh0038	p-Cresol			
	wh0693	lipoic acid			

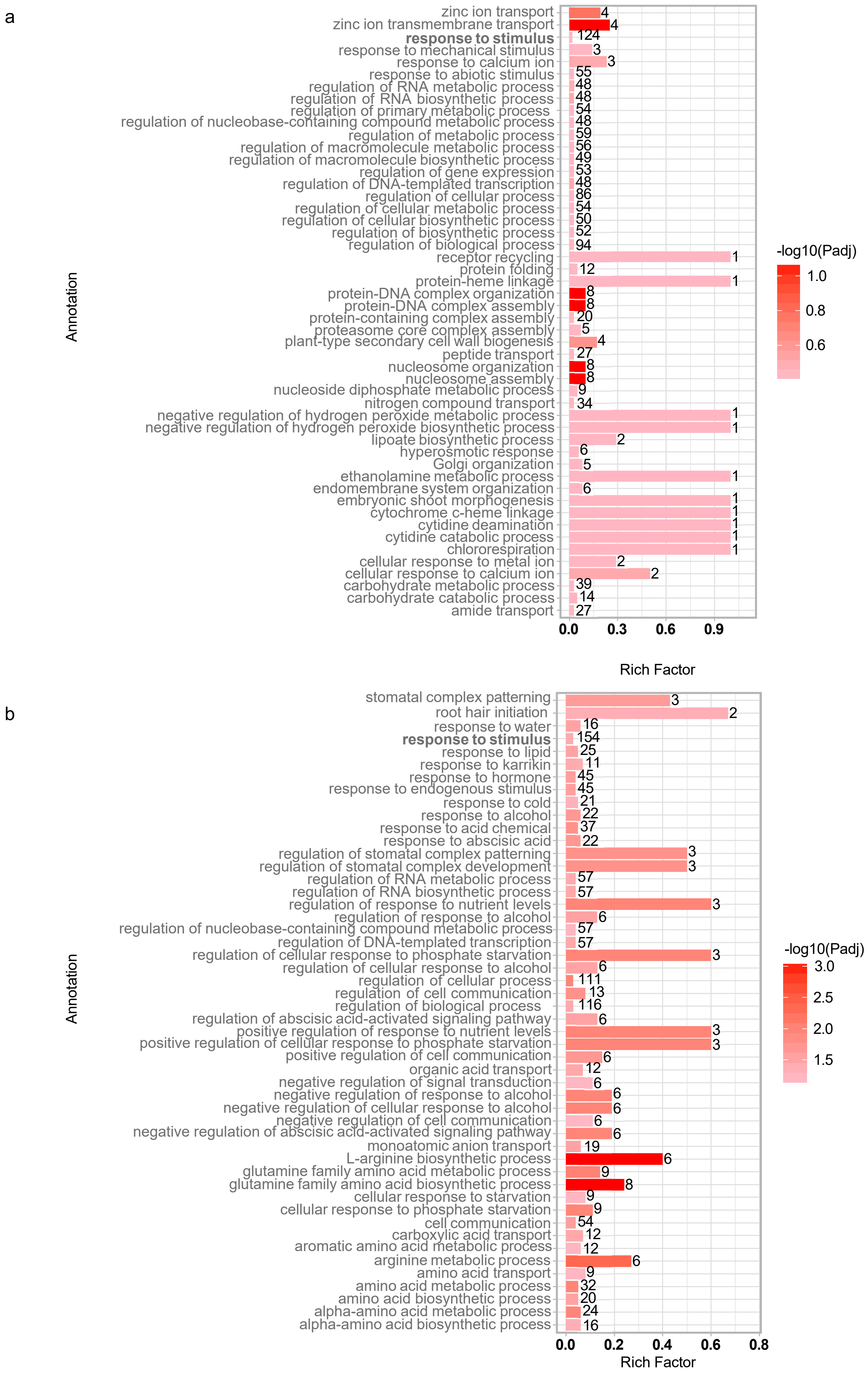

Figure S3 The significantly-enriched GO term of differentially expressed genes in the *xiaomi*. **a** GO enrichment

analysis of DEGs in Cluster 5. **b** GO enrichment analysis of DEGs in Cluster 6.
